# The effect of light emission spectrum on biohydrogen production by *Rhodopseudomonas palustris*

**DOI:** 10.1007/s00449-023-02863-8

**Published:** 2023-03-28

**Authors:** Catharine Elizabeth Bosman, Robert William McClelland Pott, Steven Martin Bradshaw

**Affiliations:** Department of Process Engineering, Stellenbosch University, Private Bag X1, Matieland, 7602 South Africa

**Keywords:** Emission spectrum, Illumination, Photobioreactor, *Rhodopseudomonas palustris*, Photofermentation

## Abstract

Photofermentative hydrogen production has gained increasing attention as a source of green energy. To make such photofermentation processes economically competitive, operating costs need to be reduced, possibly through outdoor operation. Because photofermentation processes are light dependent, the emission spectrum and intensity of light both have a significant influence on the hydrogen production and merit investigation. This study investigates the effect of light sources on the hydrogen production and growth of *Rhodopseudomonas palustris*, comparing the organism’s productivity under longer-wavelength light and light mimicking sunlight. Hydrogen production is enhanced under longer-wavelength light, producing 26.8% (± 7.3%) more hydrogen as compared to under light mimicking that of sunlight; however, *R. palustris* is still able to produce a considerable volume of hydrogen under light with a spectrum mimicking that of sunlight, providing a promising avenue for future research.

## Introduction

Purple non-sulphur bacteria (PNSB) have gained increasing attention as candidate organisms for photofermentative hydrogen production. One such PNSB species is *Rhodopseudomonas palustris* (*R. palustris*)—a photosynthetic microorganism able to produce hydrogen via the photoheterotrophic metabolic pathway, i.e. under anaerobic conditions and in the presence of a light source and suitable carbon substrate. Many different light sources have been implemented in photofermentation studies, but little work has been conducted on comparing the effects of the emission spectrum. For photofermentative hydrogen production to be economically feasible, operating costs need to be reduced. One way of doing this is through the use of natural sunlight as light source. Therefore, investigation into factors such as the effect of sunlight emission spectrum is required to determine the feasibility of outdoor operation.

The photosystem of PNSB is similar to ‘photosystem II’ found in algae and higher plants, although it does not contain ‘photosystem I’ also present in the photosystem of algae and higher plants [1]. The photosystem of PNSB comprises a reaction centre and photosynthetic units containing both carotenoid and bacteriochlorophyll *a* (BChl *a*) photosynthetic pigments. These bacterial cells have an absorption band in the visible region between 450 and 550 nm (carotenoids) as well as absorption peaks in the near-ultraviolet region at 390 nm (BChl *a*, Soret band), in the visible region at 590 nm (BCl *a*, Q band – Q_x_ shift), and in the red-infrared region at 800, 850 and 880 nm (BChl *a*, Q band – Q_y_ shift) [2], with the latter two typically coalescing into one intermediate peak between 860 and 875 nm [3, 4]. Carotenoids generally act as “photo-protection” units [2, 5], while BChl *a* pigments serve as “light-harvesting” units [2]. Consequently, several studies have reported enhanced hydrogen productivity for PNSB when exposed to illumination of longer wavelengths (red-infrared light, 750–950 nm) [4, 6]. PNSB also have longer production lag-times in the absence of red-infrared light (where BChl *a* absorbs light), whereas the absence of blue light (where carotenoids absorb light) seems to have little effect on hydrogen production and growth [4]. Sunlight provides light in all these spectral ranges and outdoor hydrogen production by PNSB has also been proven [7–10]; however, to investigate the feasibility of outdoor operation, hydrogen productivity under natural sunlight, or an artificial light source mimicking the emission spectrum of natural sunlight, should be compared to that under longer-wavelength illumination. Furthermore, little work has been done specifically on the wavelength dependence of the PNSB species *R. palustris*.

Literature-reported artificial light sources include: (i) incandescent lamps [11, 12]; (ii) halogen lamps [13, 14]; (iii) fluorescent lamps [6, 15]; (iv) Gro-Lux™ fluorescent lamps [16, 17]; and, (v) light-emitting diodes (LED) [12, 17, 18]. Incandescent and halogen lamps emit a continuous wavelength spectra, mainly in the infrared and far-infrared regions [16]. Aforementioned also most closely resemble the solar emission spectrum in the range 500–1000 nm, where *R. palustris* absorbs light. Fluorescent and LED lamps produce narrow wavelength ranges—essentially large peaks as opposed to continuous spectra [19]. When comparing white and infrared LEDs, and incandescent lamps all at the same light intensity, infrared LEDs have been shown to be most suitable for hydrogen production by *R. palustris* [12]; however, the emission spectra of LEDs do not compare well that of natural sunlight. Little work has been done on photofermentative hydrogen production under direct sunlight. Most studies on *R. palustris* under direct sunlight focus on biomass growth characteristics and photosynthetic efficiency of the bacterium rather than hydrogen production [8, 20, 21].

More knowledge is required on the effects of outdoor conditions on photofermentative hydrogen production and photofermentation systems. This study investigates the effects of the emission spectrum of light on the growth and hydrogen production of the photosynthetic bacterium *R. palustris*.

## Materials and methods

### *Bacterial species and culturing*

*Rhodopseudomonas palustris* (species NCIMB 11,774) was used in all experiments. Cells were precultured in a fast-growing medium—van Niels medium containing (per 1 L): 1 g K_2_HPO_4_, 0.5 g MgSO_4_ and 10 g biological yeast extract dissolved in deionised water [22]. Sterile glycerol (4 M, 10 mL) was added to the medium aseptically after autoclaving (121 °C, 20 min) [22]. Cells were cultured anaerobically (argon atmosphere) in 500 mL Schott bottles (~ 5 days), at 35 °C (± 0.2 °C) and under incandescent illumination (200 W·m^−2^ ± 20 W·m^−2^; Eurolux©, South Africa) [14].

Experiments were conducted in a *Rhodospirillaceae* medium (containing per 1 L): 0.6 g K_2_HPO_4_, 1.7 g KH_2_PO_4_, 0.02 g MgSO_4_·7H_2_O, 0.005 g CaCl_2_·2H_2_O, 0.4 g NaCl, 0.3 g Na_2_S_2_O_3_, 0.0005 g ferric citrate, 0.0002 g para-aminobenzoic acid, and 1 mL of trace element solution (containing per 1 L): 70 mg ZnCl_2_, 100 mg MnCl_2_·4H_2_O, 60 mg H_3_BO_3_, 200 mg CoCl_2_·6H_2_O, 20 mg CuCl_2_·2H_2_O, 20 mg NiCl_2_·6H_2_O, and 40 mg NaMoO_4_·2H_2_O [23]. After autoclaving (121 °C, 20 min), 1 mL of a filter-sterilised vitamin solution (containing per 1 L): 1.2 g thiamine HCl and 0.01 g cyanocobalamin, and 10 mL of sterile 5 M glycerol (final concentration of 50 mM), together with 5 mL of sterile glutamic acid (final concentration of 10 mM) were added to the medium aseptically[23].

### Experimental setup

#### Test-tube photobioreactor system

A test-tube reactor setup was used for all growth and hydrogen production experiments. This comprised a magnetic stirring plate, glass test-tubes (72 mL) with magnetic stirring bars immersed in a temperature-controlled glass water bath, maintained at 35 °C (± 0.2 °C). The system had one-sided illumination—halogen floodlights or ‘sunlike’ LED strips aligned with the test-tube reactors. Light intensity on the inner wall of the test-tube reactors was calibrated to 250 W·m^−2^ (± 20 W·m^−2^) in the spectral range of 450–1100 nm. Liquid samples were taken from a stainless-steel sampling port in the gastight lid on the test-tube reactors. Evolved gas was collected from a second stainless-steel port in the lid, connected to an inverted measuring cylinder immersed in a water bath (via low hydrogen-permeability tubing – Tygon E3606, Saint Gobain, South Africa). The volume of gas was monitored using the water-displacement technique.

#### Light sources

**Fig. 1:**
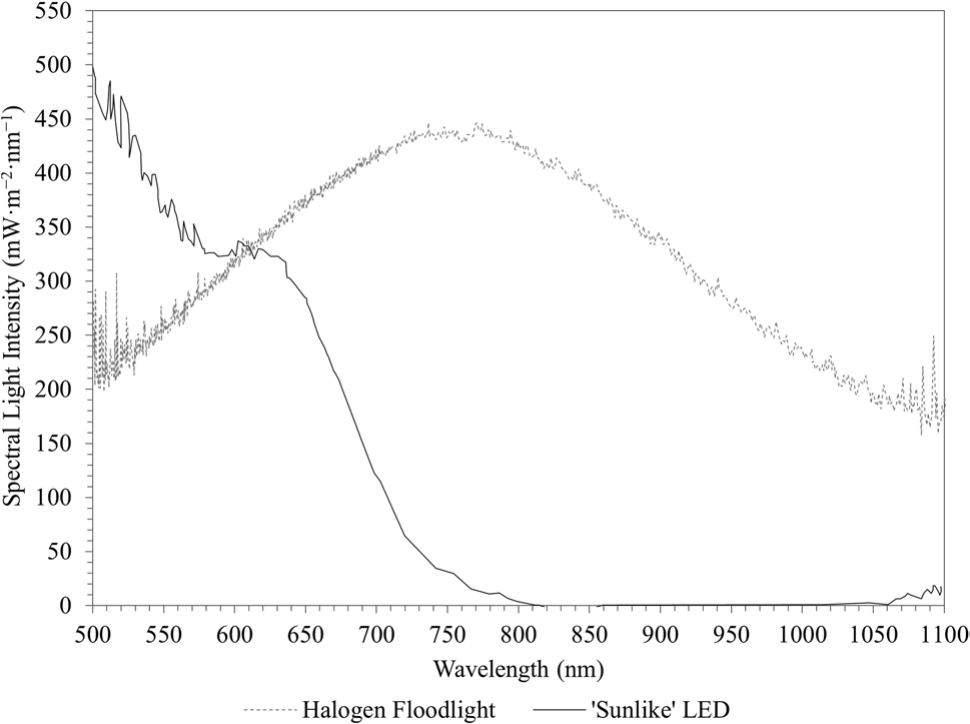
Measured emission spectra of a halogen floodlight and a ‘sunlike’ LED (250 W·m^−2^ ± 20 W·m^−2^)

*R. palustris* has been shown to produce hydrogen under halogen illumination [14], presumably due to the presence of near-infrared light. Hydrogen productivity and growth of *R. palustris* were investigated under illumination emitting wavelengths extending into the near-infrared region and illumination with an emission spectrum resembling natural sunlight (Fig. 1). For the longer-wavelength light source, halogen floodlights (Eurolux ©, South Africa, FS13, 150 W) were selected, at a colour temperature of 2900 K. For the sunlight-mimicking light, a ‘sunlike’ LED strip (LUMITRONIX LED-Technik GmbH, Germany, LumiFlex700 Pro LED Strip, 24 V) was selected, at a colour temperature of 5000 K—similar to that of natural sunlight. The emission spectrum of the LUMITRONIX LumiFlex700 ‘sunlike’ LED strip provided a good resemblance of natural sunlight in the region of approximately 500–800 nm; however, it slightly under-represented the light emitted in the near-infrared region. It should also be noted that the light emitted by both the halogen floodlight as well as the ‘sunlike’ LED slightly extends to wavelengths shorter than 500 nm; however, due to the measuring capabilities of the handheld spectrometer used in this study, the spectrum was only measured between the wavelengths of 500 and 1100 nm—beyond these wavelengths, too much noise was captured. Nonetheless, this is the wavelength range within which *R. palustris* absorbs light (500–1100 nm); therefore, this was not a problem.

### Experimental procedure

A specified concentration of precultured *R. palustris* was inoculated into the sterile *Rhodospirillaceae* medium (with glycerol). The cultures were aseptically transferred to the autoclaved (121 °C, 20 min) test-tube reactors. An anaerobic atmosphere was induced through sparging (10 min) with filter-sterilised (Midisart^®^ 2000 PTFE filter, diameter of 50 mm, pore size of 0.2 µm) argon gas (purity of > 99.9%). The test-tube reactors were immersed in a water bath pre-equilibrated to 35 °C (± 0.2 °C). Experimental runs were started by switching on the light sources, calibrated to 250 W·m^−2^ (± 20 W·m^−2^). Both liquid (biomass and glycerol concentration) and gas (gas volume) sampling were done in intervals of 24 h for a duration of 192 h. Experiments were done in quadruple to allow for the calculation of standard deviation and statistical analysis. Statistical significance was determined through *t*-tests (two-tail), by evaluating the calculated *p*-values (*p*)—number of samples (*n*) = 4, and significance level (*α*) = 0.05.

### Analytical methods

Biomass concentration was calculated using a cell dry weight (CDW) *vs*. optical density (OD) standard curve—the OD of liquid samples were measured using a UV/Vis-spectrophotometer (Model AE-S60-4U) and converted to CDW via the following correlations: CDW = 0.7126 × OD_660nm_ – 0.007; *R*^2^ = 0.9981 (Van Niels medium) and CDW = 0.6391 × OD_660nm_ + 0.0619; *R*^2^ = 0.9996 (*Rhodospirillaceae* medium) [14]. Glycerol concentration was analysed by injecting filtered samples (FilterBio^®^ Nylon Syringe Filter, 13 mm diameter, 0.22 µm pore size) into a high-performance liquid chromatograph (Dionex UltiMate 3000 HPLC). The composition of the evolved gas was analysed with a packed column gas chromatograph (Global Analyser Solutions Compact Gas GC), fitted with a thermal conductivity detector. The composition of the evolved gas was determined to be 92% (± 1.9%) hydrogen and CO_2_ the balance—similar to the compositions reported in literature (88–94% [14], 93–96% [24] and 97% H_2_ [11]).

## Results and discussion

**Fig. 2:**
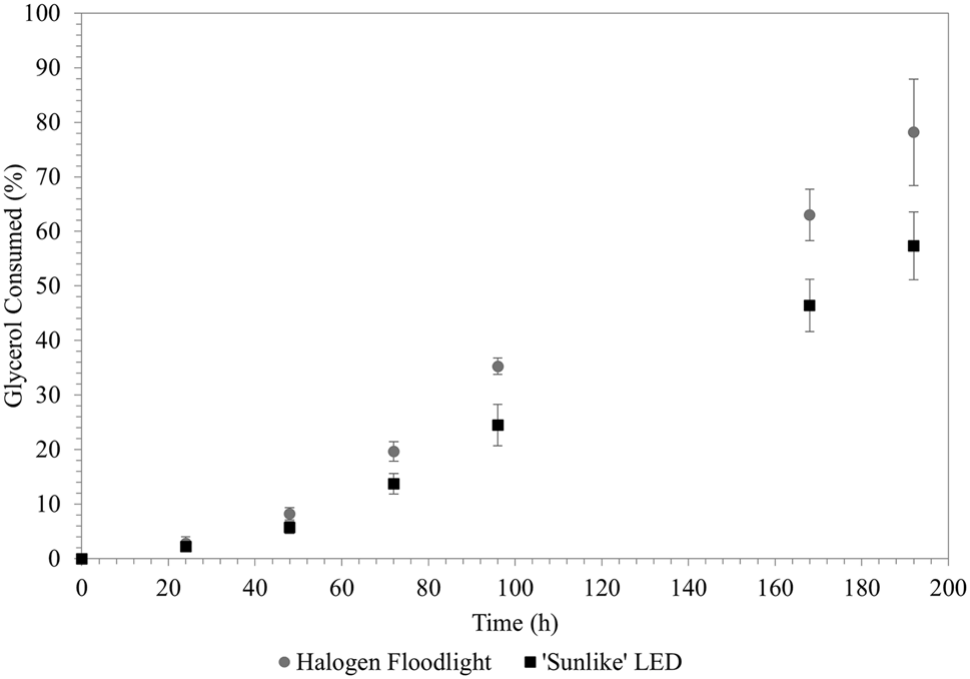
Time-dependent glycerol consumption by *R. palustris* under ‘sunlike’ LED and halogen illumination (*R. palustris* cultured in *Rhodospirillaceae* medium at 35 ºC; each data point represents the mean of 4 repeats)

The effects of the light sources and their emission spectra were investigated in terms of glycerol consumption, hydrogen production and microbial growth. Under LED illumination, *R. palustris* consumed 57.4% (± 6.2%) of the glycerol substrate, while 78.1% (± 9.7%) was consumed under halogen illumination—this is a 37.1% (± 18.6%; *p* = 0.016) increase from LED illumination to halogen illumination (Fig. 2). Under both light sources, an almost linear correlation was observed between glycerol consumption and time within the time period of 48–192 h, with the glycerol being consumed slightly faster under halogen illumination.

The hydrogen yield was determined as the molar ratio of hydrogen produced (at NTP) to the theoretical maximum of glycerol consumed according to the stoichiometric conversion of glycerol to hydrogen: C_3_H_8_O_3_ + 3H_2_O → 3CO_2_ + 7H_2_. Hydrogen yields of 19.0% (± 2.3%) and 17.6% (± 1.6%) were determined for the LED and the halogen systems, respectively, with no statistically significant difference between the yields of the two different lighting systems (*p* = 0.374). The hydrogen yields were comparably lower than other literature reported yields for systems using *R. palustris* to convert glycerol to hydrogen – 45–77% in a thermosiphon photobioreactor under halogen illumination [14] and ~ 90% in Schott bottle photobioreactors under incandescent illumination [11]. The molar ratios of hydrogen gas produced per glycerol consumed are provided in Table 1—as for the hydrogen yield, there were no statistically significant differences between the two different illumination systems (*p* = 0.210). However, these ratios were slightly lower than literature reported ratios for *R. palustris* systems operating under incandescent illumination, i.e*.* approximately 4.6–6.3 mol of H_2_ per 1 mol of glycerol substrate [11, 22]. The maximum hydrogen to glycerol ratios were achieved after approximately 96 h of operation, after which the ratios started to decrease. This suggests that more of the glycerol was utilised for hydrogen production in the times up to 96 h, while more of the glycerol was being diverted to metabolic pathways other than hydrogen production after 96 h, e.g*.* the production of internal storage products.

**Table 1:** Molar ratio of hydrogen gas produced over glycerol substrate consumed under ‘Sunlike” LED and halogen illumination, respectively

Time	H_2_ produced/Glycerol consumed (mol·mol^−1^)	
	‘Sunlike’ LED	Halogen
Maximum (after 96 h)	2.70 (± 0.46)	2.34 (± 0.16)
Overall (after 192 h)	1.33 (± 0.16)	1.23 (± 0.11)

**Fig. 3:**
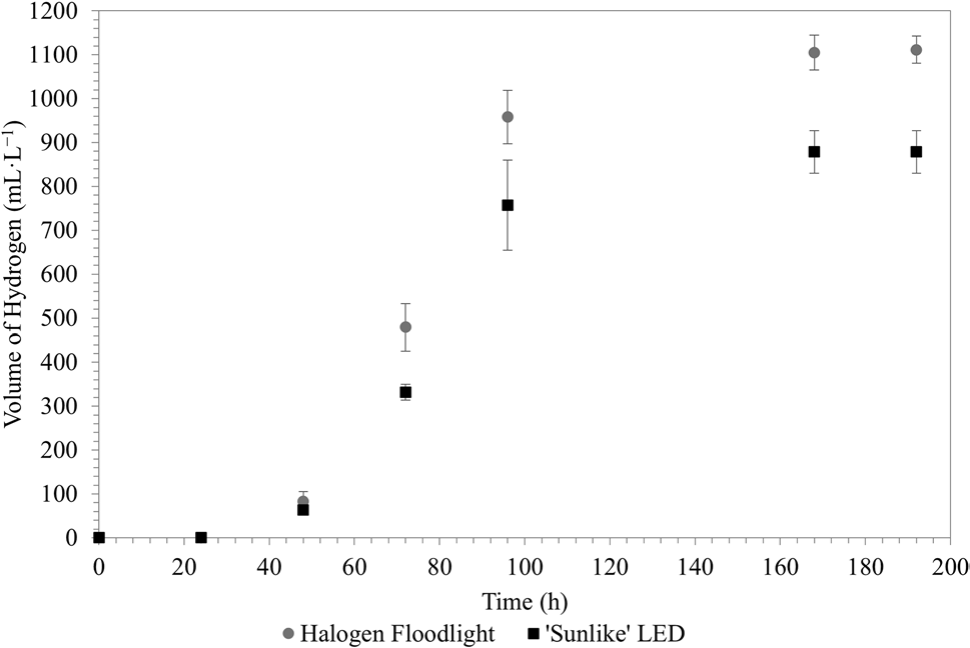
Cumulative volume of hydrogen produced by *R. palustris* per reactor working volume, under ‘sunlike’ LED and halogen illumination (*R. palustris* cultured in *Rhodospirillaceae* medium at 35 ºC; each data point represents the mean of 4 repeats)

Both illumination systems demonstrated a hydrogen production lag phase between approximately 0 and 48 h with very little hydrogen being produced during this period. The lag phase was followed by an almost linear correlation between hydrogen production and time, similar to that observed for glycerol consumption. After the linear increase, a cessation of hydrogen production was observed for both systems after approximately 96 h. Hydrogen production rates (over 192 h) of 0.204 mol·m^−3^·h^−1^ (± 0.011 mol·m^−3^·h^−1^) and 0.258 mol·m^−3^·h^−1^ (± 0.007 mol·m^−3^·h^−1^) were determined for the LED and the halogen systems, respectively. The maximum hydrogen production rates achieved for the ‘sunlike’ LED and halogen illumination systems were 0.790 mol·m^−3^·h^−1^ ± 0.158 mol·m^−3^·h^−1^ (72–96 h) and 0.891 mol·m^−3^·h^−1^ ± 0.013 mol·m^−3^·h^−1^ (72–96 h), respectively. The system under halogen illumination achieved a significantly higher hydrogen production rate (*p* = 0.0005) with a 26.8% (± 7.3%; *p* = 0.0005) increase in the total volume of hydrogen produced over the course of the experimental runs (Fig. 3). This corroborates the findings by Nogi [6] on the wavelength dependence of the PNSB *Rhodopseudomonas rutila*, stating that light of longer wavelengths (> ~ 500 nm) had a more significant effect on the biohydrogen production of the organism than light at shorter wavelengths. Similar findings were also reported for the PNSB *Rhodobacter sphaeroides* [4], where the absence of infrared light negatively affected hydrogen production, while the absence of blue light had no major effect [4]. Higher volumes of evolved hydrogen and hydrogen production rates were to be expected under halogen illumination, as more light is emitted at longer wavelengths in the near-infrared region where the BChl* a* photosynthetic pigments of *R. palustris* absorb light. As shown in Fig. 1, the emission spectrum of the ‘sunlike’ LED light emits little to no light in the near-infrared region, with the emission spectrum consisting mostly of visible light, where carotenoids absorb light. In the event of *R. palustris* or PNSB being exposed to light with shorter wavelengths, i.e*.* little to no light in the near-infrared region, the photosystem of these cells generally adjusts to the emission spectrum of the light by producing more BChl* a* pigments to more effectively absorb the little light in the desired wavelengths [4]. Although the ‘sunlike’ LED light used in this study provides a close resemblance of natural sunlight, and therefore some valuable insight into the behaviour of *R. palustris* under natural outdoor conditions, it should be noted that natural sunlight provides light in the entire wavelength spectrum and not only in the visible region like the LEDs used here. Thus, photofermentative hydrogen production should presumably be enhanced under natural sunlight as compared to under the ‘sunlike’ LEDs. Under direct sunlight, slightly higher hydrogen production rates and substrate to hydrogen gas conversions have been reported for *R. palustris*; however, this was achieved using a different substrate, bacterial strain and a different PBR system [7]—all factors which influence the productivity of photofermentative hydrogen production systems (Table 2).

**Table 2:** Comparison of hydrogen productivity between the current study under artificial illumination and a study using *R. palustris* under direct sunlight

Illumination source	‘Sunlike’ LED	Direct sunlight
Photobioreactor	Test-tube PBR (72 mL)	Tubular PBR (50 L)
Strain	*R. palustris* NCIMB 11774	*R. palustris* 420 L
H_2_ Production Rate (mol·m^−3^·h^−1^)	0.790 (± 0.158)	1.2
Substrate to H_2_ Conversion	2.70 (± 0.46) mol H_2_/1 mol glycerol	2.98 mol H_2_/1 mol malate
Reference	Current study	Adessi et al*.* [7]

With regards to outdoor operation, the findings in this study suggest that PBRs be oriented in such a way to receive most of its light during the mornings and evenings, as most longer-wavelength light is emitted during these periods. Furthermore, in the event of continuous illumination being implemented, *i.e.* sunlight during the day and artificial illumination during the night, it is also recommended that artificial light sources emitting light in the near-infrared region be implemented to achieve optimal hydrogen production.

**Fig. 4:**
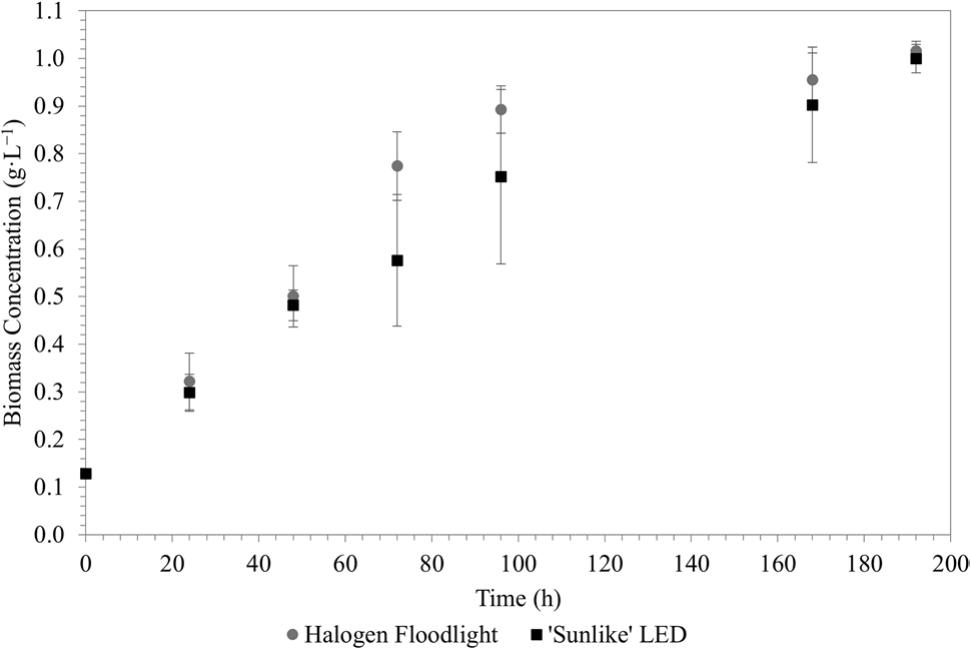
*R. palustris* biomass concentration under ‘sunlike’ LED and halogen illumination as a function of time (*R. palustris* cultured in *Rhodospirillaceae* medium at 35 ºC; each data point represents the mean of 4 repeats)

Microbial growth under the ‘sunlike’ LED seemed to be slightly slower than under the halogen illumination; however, at the end of the experimental runs at 192 h, there were no discernible difference in the final concentrations of *R. palustris*—approximately 1.0 g·L^−1^ for both systems (Fig. 4). This suggests that *R. palustris* has the ability to grow on both shorter wavelength light (< ~ 500 nm) as well as longer-wavelength light (> ~ 500 nm). This also agrees with the findings of a study on the wavelength dependence of *Rhodobacter sphaeroides* [4].

## Conclusions

The growth and hydrogen productivity of *R. palustris* were investigated under halogen illumination with an emission spectrum extending into the near-infrared region and under LED illumination with an emission spectrum mimicking that of natural sunlight. Hydrogen production was enhanced under the longer-wavelength light emitted by the halogen light source; however, the hydrogen yield remained relatively constant under the two different light sources. No pronounced effect was observed on the growth of *R. palustris*.

Although the hydrogen productivity of *R. palustris* was slightly better under halogen illumination as compared to the ‘sunlike’ LED illumination, the difference in production volumes and rates were not as pronounced, suggesting that PBRs with *R. palustris* should be able to operate under light with the emission spectrum of natural sunlight; however, hydrogen production could be further enhanced by the addition of longer-wavelength artificial illumination during night-time periods.

## Data Availability

The datasets generated and/or analysed during the current study are available from the corresponding author on reasonable request.

## References

[1] Roszak AW, Howard TD, Southall J (2003). Crystal structure of the RC-LH1 core complex from *Rhodopseudomonas palustris*. Science (80-).

[2] Adessi A, De Philippis R (2014). Photosynthesis and hydrogen production in purple non-sulphur bacteria: fundamental and applied aspects. Adv Photosynth Respir.

[3] Ritchie RJ (2013). The use of solar radiation by the photosynthetic bacterium, *Rhodopseudomonas palustris*: Model simulation of conditions found in a shallow pond or a flatbed reactor. Photochem Photobiol.

[4] Uyar B, Eroǧlu I, Yücel M (2007). Effect of light intensity, wavelength and illumination protocol on hydrogen production in photobioreactors. Int J Hydrogen Energy.

[5] Muzziotti D, Adessi A, Faraloni C (2017). Acclimation strategy of *Rhodopseudomonas palustris* to high light irradiance. Microbiol Res.

[6] Nogi Y, Akiba T, Horikoshi K (1985). Wavelength dependence of photoproduction of hydrogen by *Rhodopseudomonas rutila*. Agric Biol Chem.

[7] Adessi A, Torzillo G, Baccetti E, De Philippis R (2012). Sustained outdoor H_2_ production with *Rhodopseudomonas palustris* cultures in a 50 L tubular photobioreactor. Int J Hydrogen Energy.

[8] Carlozzi P, Pushparaj B, Degl’Innocenti A, Capperucci A (2006). Growth characteristics of *Rhodopseudomonas palustris* cultured outdoors, in an underwater tubular photobioreactor, and investigation on photosynthetic efficiency. Appl Microbiol Biotechnol.

[9] Androga DD, Özgür E, Gündüz U (2011). Factors affecting the longterm stability of biomass and hydrogen productivity in outdoor photofermentation. Int J Hydrogen Energy.

[10] Boran E, Yücel M, Gündüz U, Eroǧlu I (2012). Biohydrogen production by *Rhodobacter capsulatus* Hup- mutant in pilot solar tubular photobioreactor. J Hydrog Energy.

[11] Pott RWM, Howe CJ, Dennis JS (2013). Photofermentation of crude glycerol from biodiesel using *Rhodopseudomonas palustris*: Comparison with organic acids and the identification of inhibitory compounds. Bioresour Technol.

[12] Xiao N (2017) Use of a Purple Non-Sulphur Bacterium, *Rhodopseudomonas palustris*, as a Biocatalyst for Hydrogen Production from Glycerol (Doctoral Dissertation). University of Cambridge, Cambridge

[13] Bosman CE, Pott RWM, Bradshaw SM (2022). Design, modelling and simulation of a thermosiphon photobioreactor for photofermentative hydrogen production. Biochem Eng J.

[14] Bosman CE, Pott RWM, Bradshaw SM (2022). A thermosiphon photobioreactor for photofermentative hydrogen production by *Rhodopseudomonas palustris*. Bioengineering.

[15] Ogbonna JC, Soejima T, Tanaka H (1999). An integrated solar and artificial light system for internal illumination of photobioreactors. J Biotechnol.

[16] Carvalho AP, Silva SO, Baptista JM, Malcata FX (2011). Light requirements in microalgal photobioreactors: an overview of biophotonic aspects. Appl Microbiol Biotechnol.

[17] Kommareddy A, Anderson G (2003) Study of light as a parameter in the growth of algae in a photo-bio reactor (PBR). In: ASAE annual international meeting

[18] Niño-Navarro C, Chairez I, Christen P (2020). Enhanced hydrogen production by a sequential dark and photo fermentation process: effects of initial feedstock composition, dilution and microbial population. Renew Energy.

[19] Bertling K, Hurse TJ, Kappler U, Rakic AD (2006). Lasers—an effective artificial source of radiation for the cultivation of anoxygenic photosynthetic bacteria. Biotechnol Bioeng.

[20] Carlozzi P, Sacchi A (2001). Biomass production and studies on *Rhodopseudomonas palustris* grown in an outdoor, temperature controlled, underwater tubular photobioreactor. J Biotechnol.

[21] Wang Q, Shen L, Zhao Z (2018). Efficient culture of *Rhodopseudomonas palustris* using landfill leachate. J Pure Appl Microbiol.

[22] du Toit JP, Pott RWM (2021). Heat-acclimatised strains of *Rhodopseudomonas palustris* reveal higher temperature optima with concomitantly enhanced biohydrogen production rates. Int J Hydrogen Energy.

[23] Pott RWM, Howe CJ, Dennis JS (2014). The purification of crude glycerol derived from biodiesel manufacture and its use as a substrate by *Rhodopseudomonas palustris* to produce hydrogen. Bioresour Technol.

[24] Bosman CE, Pott RWM, Bradshaw SM (2023). Modelling and testing of a light reflector system for the enhancement of biohydrogen production in a thermosiphon photobioreactor. J Biotechnol.

